# Sleep‐inducing effect of *Passiflora incarnata L.* extract by single and repeated oral administration in rodent animals

**DOI:** 10.1002/fsn3.1341

**Published:** 2019-12-19

**Authors:** Gwang‐Ho Kim, Yehlim Kim, Sunmi Yoon, Sung‐Jo Kim, Sun Shin Yi

**Affiliations:** ^1^ Department of Biomedical Laboratory Science College of Medical Sciences Soonchunhyang University Asan Korea; ^2^ Department of Biotechnology Hoseo University Asan Korea

**Keywords:** GABA receptors, immobility, insomnia, palpebral closing time, *Passiflora incarnata L.*

## Abstract

Social cost of insomnia in modern society is gradually increasing. Due to various social phenomena and lifestyles that take away the opportunity of good quality of sleep, problems of insomnia cannot be easily figured out. Prescription of sleeping pills for insomnia patients can cause other inconveniences due to their side effects beyond their intended purposes. On the other hand, *Passiflora incarnata L.* (PI) has been widely used in South America for several centuries, showing effectiveness for sleep, sedation, anxiety, and so on in the civilian population. However, reports on the treatment efficacy of this herbal medicinal plant for insomnia patients through standardization as a sleeping agent have been very rare. Therefore, we obtained leaves and fruits of PI (8:2 by weight) as powder to prepare an extract. It was then applied to C6 rat glioma cells to quantitate mRNA expression levels of GABA receptors. Its sleep‐inducing effect was investigated using experimental animals. PI extract (6 μg/ml) significantly decreased GABA receptors at 6 hr after treatment. Immobility time and palpebral closing time were significantly increased after single (500 mg/kg) or repeated (250 mg/kg) oral administration. In addition, blood melatonin levels were significantly increased in PI extract‐treated animals after both single and repeated administrations. These results were confirmed through several repeated experiments. Taken together, these results confirmed that PI extract had significant sleep‐inducing effects in cells and animals, suggesting that PI extract might have potential for treating human insomnia.

## Graphical abstract

Primers used for quantitative real‐time polymerase chain reaction analysis for GABA receptors and related genes.

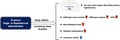

## 1 INTRODUCTION

Many people today are complaining of sleep deprivation. The number of people with insomnia is increasing worldwide. It is very uncomfortable for insomnia patients as many existing sleep treatment agents might have harmful effects on the body in terms of mental health (Hahn, Wang, Andel, & Fratiglioni, 2014). Abnormal sleeping conditions controlled by the circadian rhythm can have a very bad effect on people's quality of life, various metabolic mechanisms in the body (Elbaz et al., 2017), and brain functions (Backhaus et al., 2006; Fulda & Schulz, 2001; Gorgoni et al., 2013; Joo, Kim, Suh, & Hong, 2014). In addition, there are many environmental factors that lead to sleep deprivation in modern people's lives (Passos et al., 2014; Te Lindert et al., 2018). Because of the importance of sleep, many prescription drugs are being continuously introduced into the market. However, many of these drugs have side effects on the body such as hyperphagia, obesity, diabetes, bad mood, and unrecognizing abnormal behaviors that can cause problems besides their effects of inducing sleep (Magee, Huang, Iverson, & Caputi, 2010). Meanwhile, oriental herbal medicine has recently attracted attention as alternative medicine for treating insomnia that can help relieve hypertension, improve cognitive function, and promote sleep induction while having increased safety and efficiency. In particular, *Passiflora incarnata L.* (PI), also known as passion flower, has been highlighted due to its safe and promising sedative, sleep‐inducing, and anti‐anxiety effects (Aoyagi, Kimura, & Murata, 1974; Jawna‐Zboinska et al., 2016; Kim, Lim, Lee, & Kim, 2017; Ngan & Conduit, 2011; Yurcheshen, Seehuus, & Pigeon, 2015). It has been reported that *P. incarnata* possesses several CNS‐depressing effects. Thus, many bioactive metabolites of PI have been experimentally explored (Diniz et al., 2015), showing promising safety and efficacy with therapeutic potential (Ahn, Ahn, Cheong, & Dela Pena, 2016).

However, to obtain safe and efficient sleep‐inducing effects of PI, standardization of lead compounds and evaluation of efficacy at certain concentrations are required with experimental animals first. For this purpose, PI extraction was standardized, and GABA receptors (GABA‐beta and GABA‐delta receptors and related gene such as GAD1) after treatment with PI extract were determined by real‐time polymerase chain reaction (PCR) using in vitro and in vivo experiments. Sleep‐inducing effect of PI extract was verified by comparing animals’ immobility and palpebral closing time after single and repeated oral administrations in the present study. Results of this study suggest that PI extract can be used as a promising sleep‐inducing agent to treat insomnia.

## 2 MATERIALS AND METHODS

### 2.1 Preparing of PI extract

PI extract was obtained from leaves and fruit of PI. The preparing method is established in our previous study. Briefly, powder of PI leaves and fruits was extracted with 60% aqueous ethanol for 4 hr. The aqueous extract was dried by vacuum evaporation. After vacuum drying, the extract was standardized using 0.1% vitexin as a reference compound.

### 2.2 HPLC analysis

PI extract powder (1 g) was dissolved in 50 ml of 50% ethanol for 10 min with sonication. Filtration was performed with a 0.45‐μm syringe filter. HPLC was performed using an Agilent 1200 System equipped with a model G1312A binary LC pump, an autosampler, and a diode‐array detector. A C‐18 (Waters, SunFire™ C18) column (250 × 4.6 mm id and 5 μm particle size) was selected. Standards were purchased from Sigma Chemicals.

Chromatographic separations were performed with a mobile phase consisting of 0.1% phosphoric acid prepared in nanopure water (87%) and 100% acetonitrile (13%) for 60 min. The injection volume was 20 μl. The mobile phase flow rate was 1 ml/min. The oven temperature was 35°C, and the detection wavelength was 360 nm.

### 2.3 Experimental animals and husbandry

Seven‐week‐old ICR mice (body weight, 24–27 g) were used as experimental animals. They had no special disease, pathogen, or genetic defects. Mice were housed at room temperature (RT) (22 ± 2°C) with 60% humidity under a 12‐hr light: dark cycle (light cycle: dark cycle from 07:00 to 19:00). They were provided free access to normal chow diet (2018S; Harlan) and distilled water. Mice were used in this study after week of acclimation. They were separated into two or three groups according to single or repeated oral PI administrations. For the present study, distilled water as vehicle (Veh) control or soluble PI extract was carefully administered once or daily for 5 days with an oral sonde to animals. Veh‐ (*n* = 8, Veh‐treated only), PI 250 (*n* = 8, 250 mg/kg of PI extract), or PI 500 (*n* = 8, 500 mg/kg of PI extract) was administered. Male ICR mice were purchased from Saeron Bio. These mice were sacrificed on the second or the 6th day after beginning the administration. Most experiments of this study were performed with these mice. However, Sprague Dawley (SD) rats were specifically employed for palpebral closing time analysis. Since the eyes of mice were too small to measure palpebral closing time, thus, we used the SD rat model as an alternative method. Male SD rats were purchased from Saeron Bio. These SD rats were housed under the same conditions as ICR mice in different rooms. SD rats were used in this study after 1 week of acclimation. They were separated into two or three groups according to single or repeated oral PI administrations. For the present study, distilled water (Veh) or soluble PI extract was carefully administered once or daily for 5 days with an oral sonde to animals. Veh‐ (*n* = 8, Veh‐treated only), PI 250 (*n* = 8, 250 mg/kg of PI extract), or PI 500 (*n* = 8, 500 mg/kg of PI extract) was administered. These mice and rats were sacrificed on the second or the 6th day after beginning the administration.

### 2.4 Immunohistochemistry staining with c‐fos antibody to evaluate action area in brain after PI extract administration

Immunohistochemical staining was conducted using protocols used in our group. Briefly, sections were sequentially treated with 0.3% hydrogen peroxide in (H_2_O_2_) PBS at RT for 30 min and 10% normal goat or rabbit serum in 0.05 M PBS at RT for 30 min. They were then incubated with diluted goat anti‐c‐fos antibody (1:500, Santa Cruz Biotechnology) at 4°C overnight and subsequently incubated with biotinylated goat anti‐goat IgG and streptavidin–peroxidase complex (diluted 1:200, Vector) at 25°C for 2 hr. Sections first underwent an overnight incubation with rabbit anti‐COX‐2 antibody (1:200, Cayman) or mouse phospho‐IκBα (1:500; Santa Cruz Biotechnology) at 4°C for 48 hr. Thereafter, sections were incubated with biotinylated goat anti‐rabbit IgG or anti‐mouse IgG and a streptavidin–peroxidase complex (1:200, Vector) at 25°C for 2 hr. Sections were then visualized by staining with 3, 3’‐diaminobenzidine in 0.1 M Tris‐HCl buffer (pH 7.2). Sections were mounted onto gelatin‐coated slides with Canada balsam (Wako) following dehydration.

### 2.5 Cell culture and reagents

Rat C6 glioma cell line (C6, ATCC‐CCl‐107) was purchased from the American Type Culture Collection (ATCC). C6 glioma cells were cultured in Dulbecco's modified Eagle's medium high glucose (DMEM, 4.5 g/L glucose; Corning) supplemented with 10% (v/v) thermal‐inactivated fetal bovine serum (Gemcell, GEMINI Bio‐Products) and 1% (v/v) penicillin–streptomycin solution (Thermo Fisher Scientific) at 37°C in a CO_2_ incubator (Thermo Fisher Scientific; 95% air and 5% CO_2_).

### 2.6 Cell viability assay

Cell viability was determined using water‐soluble tetrazolium salt‐1 (WST‐1) assay kit (DoGenBio). Rat C6 glioma cells were seeded into 96‐well plates (SPL Life Sciences) at density of 3 × 10^5^ cells/well in 100 ml of media. Cells were incubated at 37°C in a 5% CO_2_ incubator overnight and then treated by PI extract at a final concentration of 0.06 μg/ml in triplicates for 24 hr. After that, 10 ml of EZ cytox cell viability assay reagent was added to each well followed by incubation at 37°C for 2 hr in a 5% CO_2_ incubator. Finally, the optical density was measured at 450 nm using a microplate reader (Sunrise™; Tecan). All experiments were repeated at least three times. Rat C6 glioma cells were also incubated with PI extract at different concentrations (6, 0.6 and 0.06 μg/ml), and the concentration that produced 50% decrease in cell viability (inhibitory concentration [IC50]) was calculated using GraphPad PRISM version 7.0.1 (GraphPad Software).

### 2.7 Measurement of intracellular ROS

Intracellular ROS level was measured using ROS indicator fluorogenic dichlorofluorescein diacetate (H_2_DCFDA, Invitrogen). Rat C6 glioma cells were incubated at 37°C in a 5% CO_2_ incubator for 6 hr. After that, cells were treated with PI extract at final concentrations of 6, 0.6, and 0.06 μg/ml for 6 hr. DW was used as a vehicle. Cells were harvested, washed with PBS twice, and analyzed by using a Guava easyCyte Flow Cytometer (Millipore). Median fluorescence intensity (MFI) was determined using FlowJo version 10.0.7.2 (TreeStar). All experiments were repeated three times. Rat C6 glioma cells were plated and treated with PI extract the same as flow cytometry analysis. After 10 μM H_2_DCFDA was added to each plate, cells were incubated at 37°C under 5% CO_2_ condition for 30 min. After washing with PBS three times, cells were visualized using a DMi8 fluorescence microscope (Leica). Images were obtained with LAS X (Leica) and Photoshop CC 2017 program (Adobe Systems).

### 2.8 Quantitative real‐time PCR

Rat C6 glioma cells were seeded into 60‐mm plates and incubated at 37°C in a 5% CO_2_ incubator for 6 hr. Cells were treated with PI extract at a final concentration of 6 μg/ml for 6 hr using DW at the vehicle. The ICR mice were administered for 5 days (vehicle and PI (250 mg kg day^‐1^) were sacrificed, and the brains were removed immediately). The hemispheres of the brains were used for RNA extraction. Total RNA was isolated using Isol‐RNA Lysis Reagent (2302700, SPRIME). MMLV reverse transcriptase (RT001, Enzynomics) was then used to generate first‐strand complementary DNA (cDNA) with the following RNA (11 μg), 1 μl of 10 p.m. oligo (dT) primers, 1 μl of dNTP mix (2 mM each), and 0.1% (v/v) DEPC‐treated water (DB0154, Bio Basic) was added up to a final volume of 16.5 μl in a 200‐μl PCR tube (Gunster Biotech). The mixture was incubated at 42°C for 5 min and immediately cooled on ice, after which 2 μl of 10× MMLV‐RT buffer, 1 μl of MMLV reverse transcriptase, and 0.5 μl RNase inhibitor (M007, Enzynomics) were added. Finally, the mixture was incubated at 42°C for 50 min followed by an incubation at 95°C for 5 min using a SimpliAmp Thermal Cycler (Applied Biosystems). For quantitative real‐time PCR (qRT‐PCR), 5 μl of 2× real‐time PCR Master Mix including SYBR Green (DQ385‐40H, Biofact), 3 μl DW, cDNA (10 μg), and 1 μl of primers (containing 10 p.m. of each of the forward and reverse primers) designed to detect Gabbr, Gabrg, and GAD1 mRNAs were added to an optical 96‐well plate (Applied Biosystems). To amplify PCR products, PCR was performed using a StepOne Plus Real‐Time PCR System (Applied Biosystems) with the following cycling conditions: pre‐denaturing at 95°C for 10 min, 40 cycles of 95°C for 15 s, and 60°C for 1 min. Primer sequences used in this study are shown in Table 1. All experiments were repeated at least three times. Relative gene expression levels were analyzed using the 2^−∆∆Ct^ method described by Livak & Schmittgen, (2001).

**Table 1:** Primers used for quantitative real‐time PCR analysis for GABA receptors and related genes

Genes	Sequences (5’3’)	
*Gabbr*	Forward	5’‐CTC TGA ACT GCG CCA TCA GC‐3’
	Reverse	5’‐TCA CAG CTA AGC CGG TCA GG‐3’
*Gabrg*	Forward	5’‐AGG ATG CTG TTC CTG CCA GA‐3’
	Reverse	5’‐TGC AGG GTG CCA TAC TCC AC‐3’
*GAD1*	Forward	5’‐CTA CCA ACC TGC GCC CTA CA‐3’
	Reverse	5’‐TTG GAG GAC TGC CTC TCC CT‐3’
*GAPDH*	Forward	5’‐TGC MTC CTG CAC CAC CAA CT‐3’ (M = A or C)
	Reverse	5’‐YGC CTG CTT CAC CAC CTT C‐3’ (Y = T or C)

### 2.9 Immobility times by single and repeated PI extract oral administrations

ICR (Saeron Bio) mice at 7 weeks old were used to measure the immobility time of experimental animals after oral administration of PI extract. In the case of single administration, vehicle (distilled water; DW), PI 250 mg/kg (PI 250), or PI 500 mg/kg (PI 500) was administered orally to each group (*n* = 10) of mice. In the case of repeated administration, vehicle (DW) or PI 250 mg/kg (PI250) was administered orally to each group (*n* = 8) of mice for 5 days. After 30 min of oral administration, immobility time of mouse was recorded for 1 hr. Recording was performed using a digital camcorder (HDR‐CX405, SONY). After video recording, the immobility time of mouse was separated into 30 s and analyzed visually. For objectivity, two observers performed measurements randomly. If the immobility time of 30 s was 10 s, it was scored 1 point. If it was 20 or 30 s, it was scored 2 or 3 points, respectively.

### 2.10 Palpebral closing times by single or repeated PI extract oral administration

We additionally performed palpebral closing time by single or repeated PI extract oral administration for 5 days with SD rats because we had a technical problem that palpebral movements of ICR mouse could not be easily recognized by video recording. To gain additional confidence with PI administration to animals, SD rats were introduced and palpebral closing time was compared with Veh‐treated group after 5‐day repeated administration of PI extract. The measurements were taken once every other day for 5 days (1st, 3rd and 5th day). Seven‐week‐old SD rats (175–225 g, Saeron Bio) were used to measure palpebral closing time of experimental animals after oral administration of PI extract. Vehicle (DW), PI 250 mg/kg (PI 250), or PI 500 mg/kg (PI 500) was administered orally to each group (*n* = 8) of SD rats for single or repeated treatments (3 times for 5 days). After 30 min of oral administration of PI extract, palpebral movements of rats were recorded for 10 min. Recording was performed using a digital camcorder (HDR‐CX405, SONY). After video recording, the palpebral closing time of each SD rat was separated by 10 s and analyzed visually. For objectivity, two observers performed measurements randomly.

### 2.11 Data analysis

To ensure objectivity, all measurements were performed under blinded conditions by two observers per experiment under identical conditions. For quantitation of immunoreactivity, the extent of staining was measured using five sections per animal. Images of c‐fos‐immunoreactive structures were taken using a BX53 light microscope (Olympus) equipped with a digital camera (DP71, Olympus) connected to a personal computer and a monitor.

Data are presented as mean ± standard error of the mean (*SEM*) for each experimental group (Veh, PI 250, or PI 500). If necessary, differences between means were analyzed with one‐way analysis of variance or two‐tailed Student's *t* test where appropriate. All statistical analyses were performed using Prism 7 (GraphPad Software). *p* < .05 were considered statistically significant (*, *p* < .05; **, *p* < .005; ***, *p* < .0005, ****, *p* < .0001).

## 3 RESULTS

### 3.1 HPLC chromatograms obtained from PI extraction

We have already established a chromatogram of PI to reveal substances contained in this PI extract in our previous study. It contained isoorientin, orientin, vitexin, and isovitexin. These results are shown in Figure S1.

### 3.2 Physiological changes after PI extract administration

**Figure 1:**
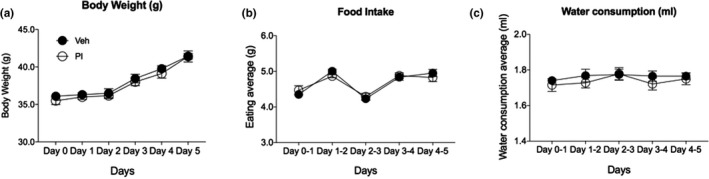
Physiological changes in mice after PI extract oral administration. (a) Body weight comparisons for 5 days after Veh‐ and PI extract administrations, (b) food intake volumes for 5 days after Veh‐ and PI extract administrations, (c) drinking water consumption in Veh‐ and PI extract‐treated group for 5 days. No factor showed any significant difference between Veh‐ and PI extract‐treated groups. Error bars represent means ± standard error of the mean (*SEM*)

For safety as a functional food, PI treatment should not affect food intake or weight changes other than the desired sleep‐inducing effect in animals. Our results revealed that body weight, food intake, or water consumption was not changed after repeated PI extract administrations (Figure 1). They showed no significant difference between PI extract‐treated animals and Veh‐treated animals. Thus, PI extract administration does not influence any basic function of animals such as eating or drinking.

### 3.3 Immunohistochemistry staining with c‐fos antibody to determine action area in the brain after PI extract administration

**Figure 2:**
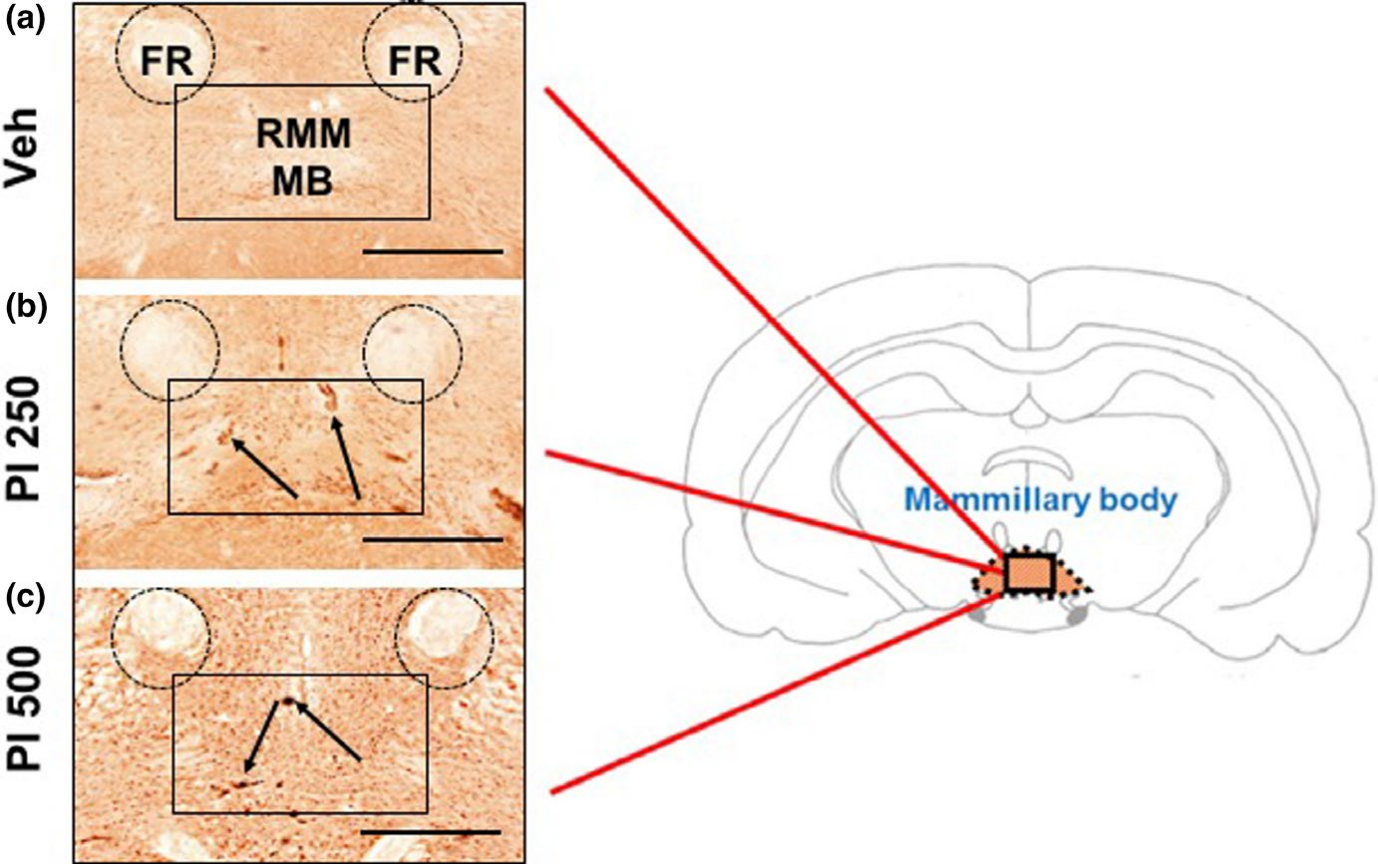
The stimulated brain area by PI extract administration in mice. Whole‐brain tissues were coronally sectioned by every 20 μm distance, and immunohistochemistry staining using anti‐c‐fos was performed. Veh (a), PI 250 (b), and PI 500 (c) images were obtained at around MB (brain diagram, the right‐sided) from bregma −2.54 mm to bregma −2.80 mm. A number of c‐fos‐positive cells are observed around the mammillary body in PI 250 and PI 500 groups, but not in Veh group. Black rectangles represent RMM of MB. MB, mammillary body; RMM, retromammillary nu medial

The primary action area in the brain after PI extract oral administration was found by immunohistochemistry staining with c‐fos antibody. As shown in Figure 2, c‐fos‐positive cells were found around mammillary body (MB) after treatment with PI 250 (250 mg/kg) or PI 500 (500 mg/kg) while there was no c‐fos signal around MB of Veh‐treated animals (Figure 2b,c). No signal was found at coronal sections of the whole cerebrum (Figure 2a).

### 3.4 ROS production and cell viability after rat C6 glioma cells were treated with PI extract

**Figure 3:**
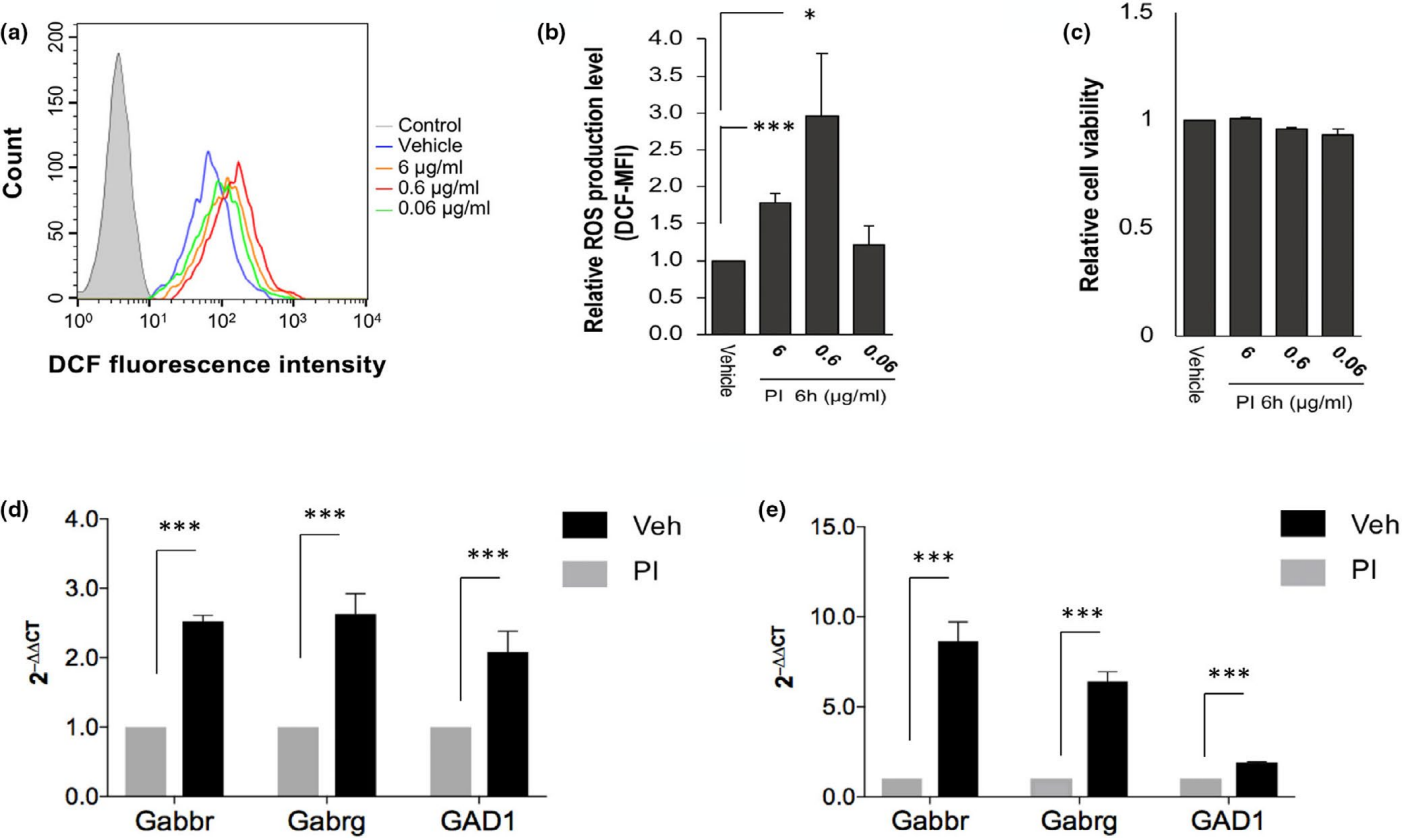
Effect of PI extract treatment for 6 hr on intracellular ROS levels and mRNA expression levels of GABA receptors. Fluorescence intensity was measured by flow cytometry. (a) Flow cytometry profiles of intracellular ROS level. (b) Quantification of relative median DCF fluorescence intensity alterations in C6 rat glioma cells treated with PI extract or vehicle. (c) All cells were treated with PI extract for 6 hr, and cell viability was determined using WST‐1 assay. The viability of C6 glioma cells was decreased significantly after treatment with PI extract at the concentration of 60 μg/ml. Others did not show any significance. (d and e) Quantification of GABA receptor mRNA expressions at the C6 glioma cells and brain hemispheres of mice by real‐time PCR after treatment with PI extract or vehicle for 6 hr and 5 days, respectively. GABA receptors and GAD1 mRNA expression levels in PI extract‐treated group were significantly higher from those in vehicle‐treated group (***, *p* < .0005; *, *p* < .05)

ROS detector H_2_DCFDA can penetrate into nonfluorescent cell membranes. When it enters the cell through the cell membrane, it reacts with esterase or HO^−^ and hydrolyzes to DCFH to remain nonfluorescent. Rat glioma cells were treated with PI extract for 6 hr prior to measuring intracellular ROS level. Cell viability was determined using WST‐1 based assay (Vistica et al., 1991), which would produce an orange formazan product that could be detected spectrophotometrically depending on mitochondrial dehydrogenase activity. Rat C6 glioma cells were incubated with PI extract for 6 hr. Relative ROS production (DCX‐MFI) values of PI extract‐treated cells at PI concentrations of 6 and 0.6 μg/ml were significantly increased (*p* < .05 and *p* < .0005, respectively, compared with those of Veh‐treated cells (Figure 3a,b). C6 glioma cells were also analyzed by WST‐1 assay for cell viability after PI extract treatment. C6 glioma cells were also analyzed by WST‐1 assay for cell viability after PI extract treatment. Results revealed that PI at concentration from 0.06 to 6 μg/ml did not significantly affect cell viability (Figure 3c).

### 3.5 Quantification of GABAa receptors and GAD1 mRNA expressions by real‐time PCR

GABAa receptors have several different subtypes such as GABA Gabbr as β‐subtype and Gabrg as γ‐subtype. Results (Figure 3d,e) showed that mRNA expression levels of Gabbr and Gabrg in PI extract‐treated group were increased compared to those in Veh‐treated group (both *p* < .0005). For Gabbr and Gabrg mRNA expression in PI extract‐treated group, its expression was significantly increased than that in the Veh‐treated group (*p* < .0005). GABA synthesis enzyme GAD1 also showed significant difference between PI extract‐treated group and Veh‐treated group (*p* < .0005).

### 3.6 Serum melatonin levels after single or repeated PI extract oral administration

**Figure 4:**
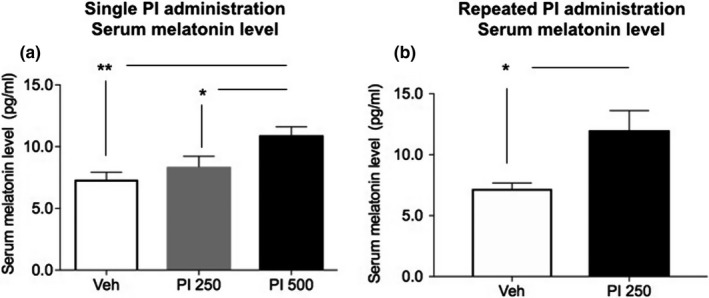
Serum melatonin levels after PI extract oral administration in mice. Blood was obtained right after animal's sacrifice, and serum melatonin level was measured. (a) Melatonin levels shown as a bar graph after a single (500 mg/kg) PI extract administration. Serum melatonin levels were significantly different between Veh‐treated and PI extract (500 mg/kg)‐treated animals (**, *p* < .01). Serum melatonin levels of PI extract‐treated animals were higher than those of Veh‐treated animals. (b) Melatonin levels shown as a bar graph after repeated PI extract (250 mg/kg) administration. Serum melatonin levels in repeated treatment group of PI extract were significantly higher than those of Veh‐treated group (*, *p* < .05). Error bars represent means ± standard error of the mean (*SEM*)

Serum melatonin level is a very critical indicator to determine whether a material has sleep‐inducing effect. Our results (Figure 4) revealed that serum melatonin levels after a single administration with vehicle or PI extract were 7.32 ± 0.612 (mean ± *SEM*) for vehicle group, 8.37 ± 0.858 pg/ml for PI 250 group, and 10.92 ± 0.694 pg/ml for PI 500 group. After repeated administrations with vehicle and PI extract (PI 250), serum melatonin levels were 7.82 ± 0.585 pg/ml and 12.00 ± 1.600 pg/ml, respectively. There were significant differences in melatonin level between vehicle control group and single administration of PI (*p* < .01 for vehicle vs. PI 250 and *p* < .05 for vehicle vs. PI 500) or repeated PI extract administration (*p* < .05).

### 3.7 Immobility time after single or repeated PI extract oral administration

**Figure 5:**
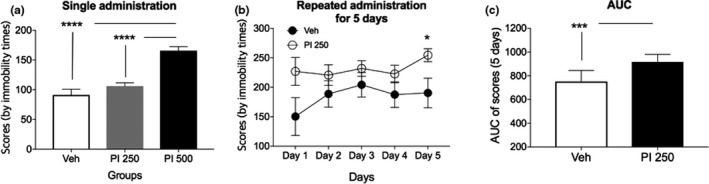
Immobility scores after single or repeated PI oral extract administration in mice. Weighted values for the length of immobility time (as immobility scores) of mice were measured for 1 hr from 30 min after oral administration of PI extract. (a) Single oral administration of PI extract was performed at concentrations of 250 and 500 mg/kg of PI extract. Immobility score of the 500 mg/kg PI extract group was significantly higher than that of the Veh‐treated group (****, *p* < .0001). However, it did not show significant difference between the 250 mg/kg of PI extract‐treated group and the Veh‐treated group, although its value was higher than that of the vehicle group. These scores were also significantly different between PI 250 and PI 500 groups (****, *p* < .00001). (b) Repeated oral administration of PI extract at concentration of 250 mg kg day^‐1^ for 5 days showed that immobility scores were higher than those of the vehicle‐treated group. However, significant difference was only obtained on the last day (Day 5) (*, *p* < .05), although immobility scores of each day were higher in the PI‐treated animal group than those of the vehicle‐treated group. (c) Area under the curve (AUC) was significantly higher in the PI‐administered group than that of Veh‐treated group (***, *p* < .0005). Error bars represent means ± standard error of the mean (*SEM*)

Immobility time of mice after single oral administered PI extract at concentration of 250 mg/kg (PI 250) or 500 mg/kg (PI 500) was prolonged compared to that of Veh‐treated mice (Figure 5a). However, it was only significantly prolonged in the PI 500 group, not in the PI 250 group, compared to that of Veh‐treated group. Immobility score of PI 500 was also significantly higher than that of PI 250 (*p* < .0001).

Immobility scores of mice after repeated oral administration of PI extract at concentration of 250 mg/kg (PI 250) for 5 days were consistently higher than those of Veh‐administered group from the beginning of administration. However, significant difference was secured only on the fifth day (Day 5) (Figure 5b; *p* < .05). Area under the curve (AUCs) also showed significant differences between PI 250 and Veh group (Figure 5c; *p* < .0005).

### 3.8 Palpebral closing time after single or repeated PI extract oral administration

**Figure 6:**
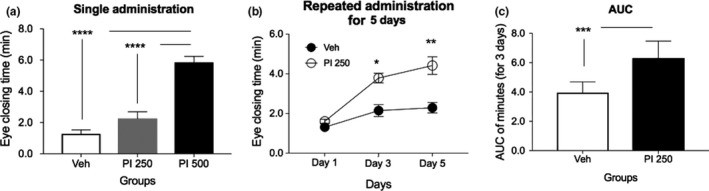
Palpebral closing time test after single or repeated PI oral extract administration in SD rats. Cumulative minutes of palpebral closing time of SD rats were measured for 1 hr from 30 min after oral administration of PI extract and vehicle. (a) Single oral administration of PI extract was performed at concentrations of 250 and 500 mg/kg. Palpebral closing time test in the 500 mg/kg of PI extract was significantly longer than that of the Veh group (****, *p* < .0001). (b) Palpebral closing time after repeated oral administration of PI extract at concentration of 250 mg kg day^‐1^ for 5 days is higher than that of Veh group. Significance differences were only obtained on the second day and the third day (Day 3 and Day 5) (*, *p* < .05; **, *p* < .005, respectively). (c) Area under the curve (AUC) was significantly higher in the PI 250‐administered group than that of Veh‐treated group (***, *p* < .0005). Error bars represent means ± standard error of the mean (*SEM*)

Palpebral closing time of SD rats after single oral administration of PI extract at concentration of 250 mg/kg (PI 250) or 500 mg/kg (PI 500) was prolonged than those of Veh‐treated SD rats (Figure 6a). Palpebral closing time of the PI 500 group was also significantly higher than that of the PI 250 group (*p* < .0001) after a single PI extract administration (Figure 6a). However, after repeated PI extract administration for five days, PI 250 had a significant difference from Day 3 between repeated Veh‐treated group at eye closing time (Figure 6b). Palpebral closing time of SD rats after repeated oral administration of PI extract at concentration of 250 mg/kg (PI 250) for 5 days (measured at Day 3 and Day 5) was consistently higher than that of the Veh‐administered group from the second day of administration. AUCs between PI 250 and Veh groups also showed significant differences (Figure 6c; *p* < .0005).

## 4 DISCUSSION

Chronic insomnia is not only a major medical problem worldwide, but also a critical problem that can seriously lower the quality of life of affected individual. Chronic insomnia not only makes affected individual feel very exhausted, but also act as an insult to the brain's microenvironment, making it uncomfortable for everyday life (Backhaus et al., 2006; Bliwise, 2004; Gagnon, Petit, Latreille, & Montplaisir, 2008; Joo et al., 2014; Trotti & Karroum, 2016). It also promotes degenerative brain disease. Although sleeping pills could be used, insomnia patients may suffer a wide variety of side effects of sleeping pills that are currently available (Park & Shin, 2016; Piran & Robinson, 2006; Schroeck et al., 2016). On the other hand, passion flowers have long been known to be effective for anxiety and insomnia in the private sector due to its sedation effect as mentioned earlier (Kim et al., 2017).

In our previous study, we have concluded that administration of PI can prevent Alzheimer's disease, increase hippocampal neurogenesis, decrease microglial population, and decrease the expression of Tau and pTau after single or repeated administration (Kim et al., 2019). Numerous studies by other research teams have consistently reported that *P. incarnata* species has sedative, anti‐anxiety, and anti‐neurodegenerative disease effects. It can also mitigate attention deficit hyperactivity disorder symptoms in children (Corona, 2018; Grundmann, Wahling, Staiger, & Butterweck, 2009; Jawna‐Zboinska et al., 2016; Ulbricht et al., 2008). However, the use of PI widely known in the private world has not been standardized for inducing sleep. If PI administration can be used as a sleep inducer for many patients who experience insomnia, it may prevent various diseases caused by chronic insomnia with a variety of benefits. In addition, according to our preliminary study, data of its effective dose accumulated through animal experiments (250 and 500 mg/kg of PI extract powder) confirmed that single (500 mg/kg) or repeated (250 mg/kg) oral administration of PI extract was effective in inducing sleep ability.

Several substances such as isoorientin, orientin, vitexin, and isovitexin were detected in the PI extract (Figure S1). Vitexin might be its major effective substance according to our result. Vitexin is considered to have the ability to induce sleepness with antidiabetic and anti‐inflammatory effects (Abbasi, Nassiri‐Asl, Shafeei, & Sheikhi, 2012; Choi et al., 2014; He et al., 2016). It is effective for sleep improvement. Therefore, functional difference between vitexin only and PI extract is worthy of further study.

PI treatment increased mRNA expressions of GABA receptors and the related genes after cells were incubated with PI for 6 hr; PI showed ability to inhibit neuronal activity (Figure 3d and e). Since high concentrations of PI were also used in the in vivo experiment, the in vitro experiment was conducted at the most valid high concentration (6 μg/ml). Interestingly, it has been reported that GABA activity is highly influenced by ROS expression (Accardi et al., 2014; Calvo & Beltran Gonzalez, 2016). The increase or decrease in the expression of ROS expression is not only related to cell damage or expression of cytokines in cells, but also acts an important factor required for the activity of GABA (Calvo & Beltran Gonzalez, 2016). Results of the present study also showed that PI extract decreased α‐subtype of GABA receptors but increased β‐ and γ‐subtypes. GABA transporters play a role in promoting GABA synthesis by increasing GAD1. Therefore, PI extract is expected to exert its efficacy by enhancing the receptivity of β‐ and γ‐subtypes to ligands and by promoting synthesis of GABA via GAD1 through some GABA transporter activation. Previous reports have shown that receptivity of β‐ and γ‐subtypes to ligands has strong contribution to synaptic transmission from GABAergic sleep‐inducing effects on hypothalamus (Kang & Macdonald, 2016; Yanovsky et al., 2012).

As mentioned earlier, there was not rapid change in animal food intake or body weight after single or repeated PI extract administration. These results were reproducible according to our experiments. In addition, through our preliminary study, we confirmed that PI had no other effects affecting metabolic activities other than its sleep‐inducing effect (data not shown). Meanwhile, the action region of PI extract treatment through the distribution of c‐fos‐positive cells is found to be retromammillary nu medial (RMM) of the MB which is known to be highly related to sleep in other reports (Fifel, Meijer, & Deboer, 2018). Therefore, it is expected that primary activity will occur at this site after injection of PI extract so that the animal can have a sleep desire. Melatonin level in the blood was increased when low concentration of PI (PI 250) was used for treatment for 1 day. However, it was significantly increased after repetitive treatment of PI (Figure 3a,b). PI 500 induced significant melatonin expression in blood when it was used for treatment only once (Figure 3a). Based on our data, the effective concentration obtained from the present study was used for a clinical study of PI which also proved that PI was effective for inducing sleep in humans (data not shown). Since many studies have reported various side effects and risks of sleeping pills based on chemicals, the introduction of PI extract in the present study is expected to provide an opportunity to minimize these side effects. This study was designed to obtain results for repeated administration of PI 250. We could not use humans as subjects because the amount of PI concentration of PI 500 needed for a single PI extract oral administration would be too large.

After PI 250 was repeatedly administered to mice, less movements of mice were confirmed (Figure 4), although the difference was not statistically significant. On the 5th day, PI‐treated mice were significantly less active than Veh‐treated ones. When AUC values were calculated for 5 days by the substance, it was found that the movement of the PI‐treated group was smaller than that of the Veh‐treated group. These results were almost the same for palpebral closing time test using SD rats (Figure 6). For consistency of these results, it may be better to experiment with mouse alone. However, since the eyelid of mouse could not be recognized properly, we had to use SD rats rather than mice for this test. In SD rats, similar to results of mice, repeated administration of PI 250 significantly increased the palpebral closing time at the measurements from 3rd and 5th day for 5 days. The value of AUC was also significant.

Our goal is to develop PI extract as a sleep‐inducing agent for human use. For this purpose, we investigated the effect of PI extract on brain environment. Metabolic and behavioral changes after repeated administration may also occur. Thus, we examined these changes. Low concentration of PI (20–50 mg/kg) increased neuronal differentiation and neurotrophic environment in the hippocampus of mice and rats. However, PI 250 or PI 500 did not cause any metabolic or behavioral changes in our previous study (data not shown). This might explain why PI has been empirically used for centuries.

## 5 CONCLUSION

Although it is impossible to explain all mechanisms by which PI extract causes sleepiness in this present study, the most basic mechanism is that it is closely related to the regulation of GABA and that its active site is limited to RMM of MB in the brain rather than multiple sites. It is a very favorable condition for the study of mechanism. Repeated ingestion of PI extract (PI 250) does not cause serious changes in body weight or abnormal feeding behaviors in animals. It has sleep‐inducing effects in rodents. PI at high concentration (PI 500) can induce sleep in mouse after a single dose. However, effective dose of PI for human would be somewhat higher. Further study is needed to determine whether PI extract can be sued as a supplementary diet for insomnia patients.

## CONFLICT OF INTEREST

The authors declare no conflict of interest.

## ETHICAL APPROVAL

All animal care and experimental protocols were ethically reviewed and approved (Approval number: SCH16‐0037) by the Soonchunhyang University Institutional Animal Care and Use Committee (IACUC). This study does not involve any human testing.
