# Supplementary figures and images for: Sleep‐inducing effect of Passiflora incarnata L. extract by single and repeated oral administration in rodent animals
Source: Food Sci Nutr. 2019 Dec 19;8(1):557–66. doi: 10.1002/fsn3.1341 (PMC6977488; doi:10.1002/fsn3.1341)

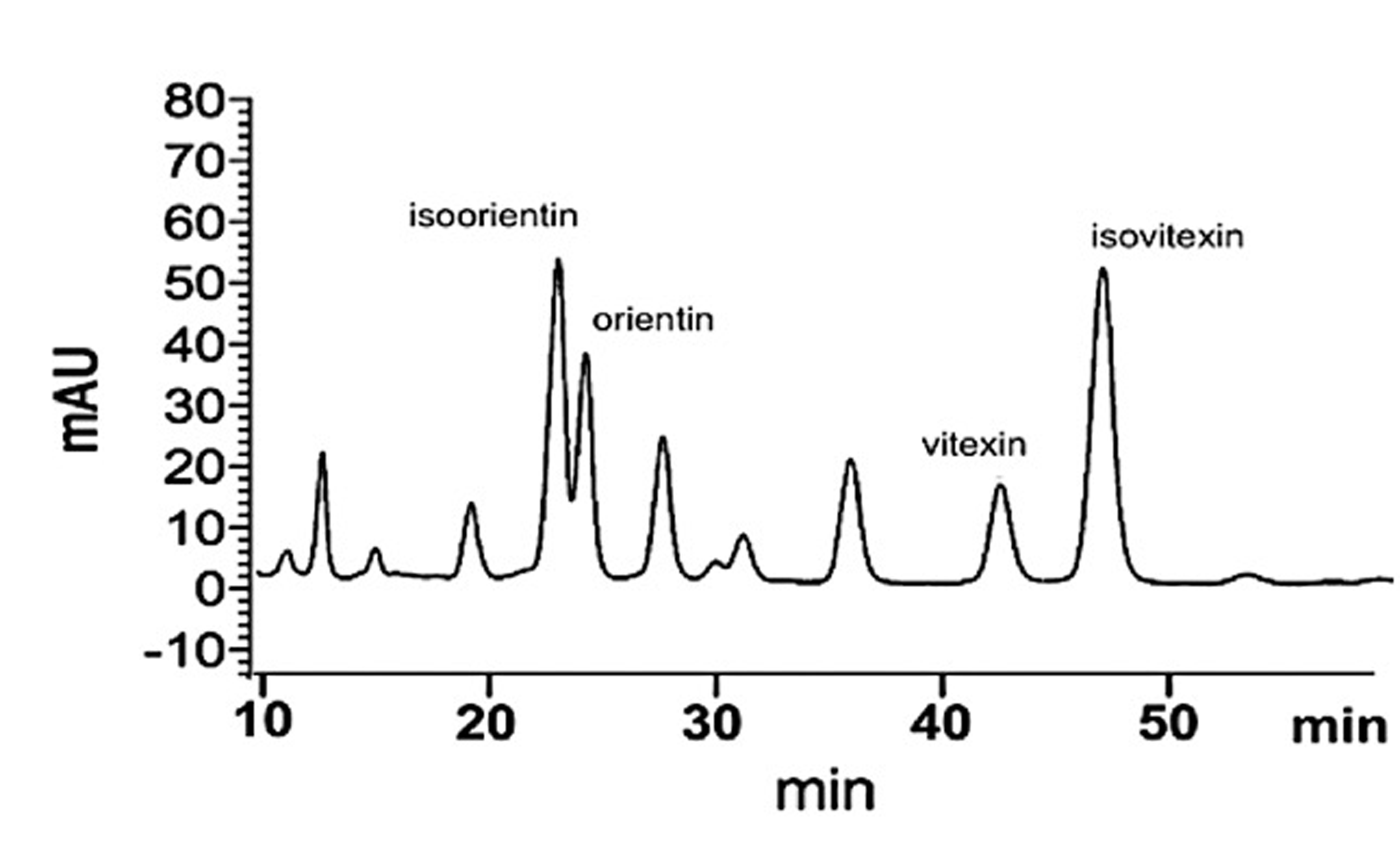

Supplement: Supplementary file 1 [file FSN3-8-557-s001.tif]
